# Ambulatory Resistant Hypertension and Risk of Heart Failure in the Elderly

**DOI:** 10.3390/diagnostics13091631

**Published:** 2023-05-05

**Authors:** Francesca Coccina, Anna M. Pierdomenico, Chiara Cuccurullo, Jacopo Pizzicannella, Oriana Trubiani, Sante D. Pierdomenico

**Affiliations:** 1 Department of Innovative Technologies in Medicine & Dentistry, University “Gabriele d’Annunzio”, Chieti-Pescara, 66100 Chieti, Italy; 2 Department of Medicine and Aging Sciences, University “Gabriele d’Annunzio”, Chieti-Pescara, 66100 Chieti, Italy; 3 Department of Engineering and Geology, University “Gabriele d’Annunzio”, Chieti-Pescara, 66100 Chieti, Italy

**Keywords:** ambulatory blood pressure, hypertension, prognosis, heart failure

## Abstract

(1) Background: The aim of the study was to assess the risk of heart failure (HF) in elderly treated hypertensive patients with white coat uncontrolled hypertension (WUCH), ambulatory nonresistant hypertension (ANRH) and ambulatory resistant hypertension (ARH), when compared to those with controlled hypertension (CH). (2) We studied 745 treated hypertensive subjects older than 65 years. CH was defined as clinic blood pressure (BP) < 140/90 mmHg and 24-h BP < 130/80 mmHg; WUCH was defined as clinic BP ≥ 140/90 mmHg and 24-h BP < 130/80 mmHg; ANRH was defined as 24-h BP ≥ 130/80 mmHg in patients receiving ≤2 antihypertensive drugs; ARH was defined as 24-h BP ≥ 130/80 mmHg in patients receiving ≥3 antihypertensive drugs. (3) Results: 153 patients had CH, 153 had WUCH, 307 had ANRH and 132 (18%) had ARH. During the follow-up (8.4 ± 4.8 years), 82 HF events occurred. After adjustment for various covariates, when compared to CH, the hazard ratio (95% confidence interval) for HF was 1.30 (0.51–3.32), 2.14 (1.03–4.43) and 3.52 (1.56–7.96) in WUCH, ANRH and ARH, respectively. (4) Conclusions: among elderly treated hypertensive patients, those with ARH are at a considerably higher risk of developing HF when compared to CH.

## 1. Introduction

A large body of evidence indicates that out-of-office blood pressure (BP), detected by ambulatory BP monitoring or home BP recording, is superior to clinic BP in predicting cardiovascular outcomes in both untreated and treated hypertensive patients [1,2,3,4,5]. Ambulatory BP monitoring gives the opportunity to obtain a lot of information including BP phenotypes in relation to clinic and ambulatory BP values [6,7]. In treated individuals receiving any number of drugs, these phenotypes include those with normal clinic and ambulatory BP, that is, controlled hypertension (CH), those with normal clinic but high ambulatory BP, that is, masked uncontrolled hypertension (MUCH), those with high clinic but normal ambulatory BP, that is, white coat uncontrolled hypertension (WUCH) and those with high clinic and ambulatory BP, that is, sustained uncontrolled hypertension (SUCH) [8,9,10,11,12,13,14,15,16,17,18,19].

Resistant hypertension occurs when treatment with ≥3 drugs fails to lower BP below upper normal limits and the unsatisfactory control of BP is confirmed by ambulatory BP monitoring or home BP recording [1,2,20,21]. In this context, patients with MUCH receiving ≤2 or ≥3 drugs may be defined as having non-resistant MUCH or resistant MUCH, respectively, and patients with SUCH receiving ≤2 or ≥3 drugs may be defined as having non-resistant SUCH or resistant SUCH, respectively, whereas patients with WUCH treated with ≤2 or ≥3 drugs may be defined as having false non-resistant and false resistant hypertension that can be considered as a unique group. In addition, from a clinical point of view, non-resistant MUCH and non-resistant SUCH can be joined in a single group named ambulatory non-resistant hypertension (ANRH), and those with resistant MUCH and resistant SUCH can be joined in a unique group termed ambulatory resistant hypertension (ARH).

Heart failure (HF) is an important public health problem characterized by high morbidity, mortality, hospitalization rates, and cost [22]. Hypertension is one of the most relevant causes of HF onset [23]. Its population-attributable risk for HF has been reported to be as high as that of coronary artery disease [24]. However, clinic BP, as for other cardiovascular outcomes, may not completely describe the detrimental effect of hypertension on HF. In this scenario, few studies have suggested that ambulatory BP may be superior to clinic BP in predicting the occurrence of HF [25,26]. Moreover, to the best of our knowledge, though the global prognostic value of ARH has been evaluated [9,27,28,29,30,31], only a single study [32] reported on the impact of ARH on the incidence of HF and no study evaluated this topic specifically in the elderly.

The aim of this study was to assess the risk of HF in elderly treated hypertensive patients with CH, WUCH, ANRH, and ARH.

## 2. Materials and Methods

### 2.1. Patients

This study is a retrospective investigation of our prospectively collected data. We included 745 treated hypertensive patients older than 65 years, the most frequent age presently used to define the progression to the elderly status in Western nations, from a cohort of 2264 sequential treated individuals with complete follow-up [33]. The global population was aged 30 to 90 years and was recruited from December 1992 to December 2012. All the patients had been referred to our hospital outpatient clinic for evaluation of BP control. Among the original population of 781 subjects older than 65 years, 36 were lost during follow-up. For the present study, the inclusion criteria were: (1) patients with hypertension; (2) use of antihypertensive medications; (3) age older than 65 years. Subjects with secondary hypertension, recent cardiovascular events (6 months), acute illness, dementia, cancer, and disability were excluded. All the subjects underwent clinical evaluation, electrocardiogram, routine laboratory tests, echocardiographic examination, and non-invasive ambulatory BP monitoring at baseline. The study population came from the same geographical area (Chieti and Pescara, Abruzzo, Italy). Our prospective observational study was approved by the Institutional Review Committee in 1992. For this specific manuscript, ethical approval was waived due to the retrospective nature of the study. Subjects gave written informed consent to be included in the study and for anonymous data processing.

### 2.2. Clinic BP Measurement

Clinic BP was detected by a physician using a mercury sphygmomanometer and appropriate-sized cuffs. Measurements were performed three times in a quiet room, 2 min apart, after at least 5 min of rest, and the mean value was used as the BP for the visit. Clinic systolic/diastolic BP < 140/90 mmHg was defined as controlled hypertension, and clinic systolic BP ≥ 140 mmHg and/or clinic diastolic BP ≥ 90 mmHg was defined as uncontrolled hypertension.

### 2.3. Ambulatory BP Monitoring

Ambulatory BP monitoring was executed with a noninvasive recorder (SpaceLabs 90207, Redmond, WA, USA) on a typical day, within 1 week from clinic evaluation. Technical aspects have been previously reported [34]. We evaluated the following ambulatory BP parameters from the examination performed at baseline: daytime (while awake as reported in the diary), nighttime (while asleep as reported in the diary), and 24-h systolic and diastolic BP, the extent of BP reduction from day to night (those with BP reduction < 10% were defined as nondippers and those with BP reduction ≥ 10% were defined as dippers), and pre-awakening morning surge of BP defined as the difference between the mean BP during the 2 h after waking and the mean BP during the 2 h before waking. Concerning morning surge of BP and nondipping pattern, we also previously categorized our elderly population into 3 groups, that is, dippers with normal morning surge of BP, dippers with a high morning surge of BP and nondippers [26]. CH was defined as clinic BP < 140/90 mmHg and 24-h BP < 130/80 mmHg; WUCH was defined as clinic BP ≥ 140/90 mmHg and 24-h BP < 130/80 mmHg; ANRH was defined as clinic BP < or ≥140/90 mmHg and 24-h BP ≥ 130/80 mmHg in patients taking ≤2 drugs (that is non-resistant MUCH and non-resistant SUCH); ARH was defined as clinic BP < or ≥140/90 mmHg and 24-h BP ≥ 130/80 mmHg in patients taking 3 or more drugs (that is resistant MUCH and resistant SUCH). Recordings were of good quality in all the subjects (at least 70% of valid readings during the 24-h period, at least 20 valid readings while awake with at least 2 valid readings per hour, and at least 7 valid readings while asleep with at least 1 valid reading per hour), according to the European Society of Hypertension requirements [35].

### 2.4. Echocardiography

A complete echocardiographic investigation, including two-dimensional, M-mode, doppler and color Doppler assessment was performed at baseline. Measurements of left atrial (LA) and left ventricular (LV) dimensions and calculation of LV mass were made according to standardized methods [36]. LA diameter (cm) was indexed by body surface area (m^2^) and LA enlargement was defined as LA diameter/body surface area ≥2.4 cm/m^2^ [36]. LV mass value was indexed by height^2.7^ and LV hypertrophy was defined as LV mass/height^2.7^ > 50 g/m^2.7^ in men and >47 g/m^2.7^ in women [37]. LV ejection fraction (EF) was computed using the Teichholz formula or the Simpson rule [36]. Asymptomatic LV systolic dysfunction at baseline was defined as an EF < 50% in patients without symptoms and signs of HF.

### 2.5. Follow-Up

Patients were followed-up in our outpatient clinic or by their general practitioner. The occurrence of events was recorded at hospitalization or during follow-up visits or by telephone interview of the general practitioner or the patient or a family member, followed by a visit if the patient was alive. Medical records were obtained to confirm the events. In the present study, we focused on HF requiring hospitalization. Diagnosis of HF was based on symptoms and signs of HF, B-type natriuretic peptide or N-terminal pro-B-type natriuretic peptide levels when available, chest X-ray and echocardiographic examination. HF subtypes were reclassified according to recent European Society of Cardiology guidelines for HF [38], that is, HF with reduced EF (≤40%), mild reduced EF (40–49%) and preserved EF (≥50%). We established a priori to analyze the different HF subtypes as a single group of HF to maintain adequate statistical power. In this study, the investigator (F.C) who evaluated the endpoint was blinded to the other patients’ data.

### 2.6. Statistical Analysis

The data are described as means ± standard deviation, median (interquartile range), or numbers and percentages. Groups were compared by using a one-way analysis of variance followed by a post hoc test (Scheffé), Kruskall–Wallis test followed by Mann–Whitney U test with Bonferroni’s correction and chi-square or Fisher’s exact test with Bonferroni’s correction, where appropriate. Event rate is expressed as the number of events per 100 patient-years based on the ratio of the observed number of events to the total number of patient-years of exposure up to the terminating event or censor. Survival curves were estimated using the Kaplan–Meier product-limit method and compared by the Mantel (log–rank) test. Univariate Cox regression analysis was used to evaluate the association of various factors, including ambulatory BP phenotypes, with outcome, calculating unadjusted hazard ratio (HR) and 95% confidence interval (CI). Then, multivariate Cox regression analysis was performed to evaluate the independent association with the outcome of WUCH, ANRH, and ARH when compared with CH, calculating adjusted HR and 95% CI. The forced entry model was used in multivariate analysis. Statistical significance was defined as *p* < 0.05. Analyses were made with the SPSS 21 software package (SPSS Inc., Chicago, IL, USA). Graphs were made with Microsoft Excel 10 (Microsoft Corporation, Redmond, WA, USA) and GraphPad Prism 7 (GraphPad Software Inc., San Diego, CA, USA).

## 3. Results

Characteristics of the studied population are reported in Table 1. We found 153 patients with CH, 153 with WUCH, 307 with ANRH (64 with non-resistant MUCH and 243 with non-resistant SUCH), and 132 (18%) with ARH (14 with resistant MUCH and 118 with resistant SUCH). Body mass index, prevalence of diabetes, reduced estimated glomerular filtration rate, LV hypertrophy, and LA enlargement were significantly different among the groups. The highest values were observed in patients with ARH.

Clinic and ambulatory BP data are reported in Table 2. Clinic, daytime, nighttime, and 24-h systolic and diastolic BP were significantly different among the groups by definition. The highest values of systolic BP were recorded in patients with ARH. Compared to patients with ANRH, daytime systolic BP tended to be higher and nighttime and 24-h systolic BP were significantly higher in patients with ARH. Morning systolic BP surge (pre-awakening), as a continuous variable, and prevalence of dippers with normal or high morning systolic BP surge were different among the groups; lower values were observed in patients with ARH. The prevalence of nondippers was also different among the groups; higher values were detected in patients with ARH.

The treatment of study groups is reported in Table 3. The use of diuretics (mainly thiazide-type), beta-blockers, calcium channel blockers, angiotensin converting enzyme inhibitors, and alpha blockers was significantly different among the groups. The above-mentioned drug classes were more frequently used in patients with ARH. Spironolactone, as a potassium-sparing diuretic, was used in 19 (14%) patients in the ARH group. Fifteen (11%) patients did not receive a diuretic because of side effects in the ARH group. Single, double, and triple therapy were different by definition. The use of aspirin and statin did not differ among the groups.

**Figure 1:**
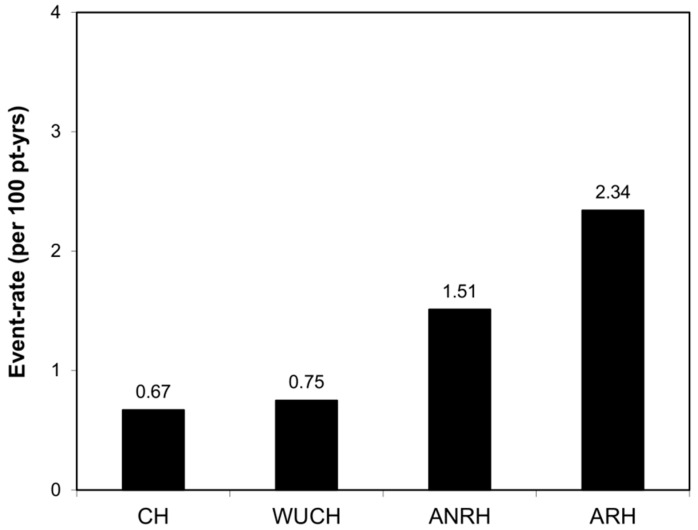
Event rate of study groups. ANRH, ambulatory non-resistant hypertension; ARH, ambulatory resistant hypertension; CH, controlled hypertension; WUCH, white coat uncontrolled hypertension.

During the course of the follow-up (8.4 ± 4.8 years, range 0.4–20.5), 82 HF events requiring hospitalization occurred, including 34 HF with reduced EF, 2 with mild reduced EF, and 46 with preserved EF (as previously stated, HF subtypes were analyzed together). None of the HF events were preceded by coronary events in this population. Specifically, there were 10, 9, 39, and 24 events in patients with CH, WUCH, ANRH, and ARH, respectively. The event rate (Figure 1) was more than twice and three times in patients with ANRH and ARH, respectively, when compared to those with CH.

**Figure 2:**
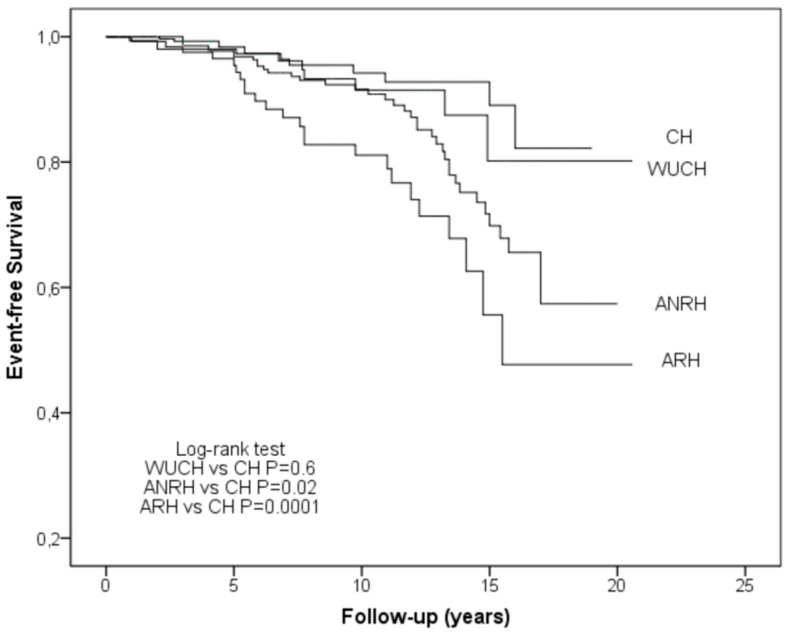
Event-free survival curves of study groups. ANRH, ambulatory non-resistant hypertension; ARH, ambulatory resistant hypertension; CH, controlled hypertension; WUCH, white coat uncontrolled hypertension.

Event-free survival curves are shown in Figure 2. Event-free survival was significantly different in patients with ANRH and ARH when compared to those with CH. Event-free survival was also significantly different in patients with ARH when compared to those with WUCH (*p* = 0.002) and ANRH (*p* = 0.026).

Univariate Cox regression analysis showed that age (HR 1.86, 95% CI 1.18–2.92, per 10 years increment), diabetes mellitus (HR 2.10, 95% CI 1.14–3.86), LV hypertrophy (HR 2.87, 95% CI 1.86–4.44) and asymptomatic LV systolic dysfunction (HR 6.49, 95% CI 3.57–11.78) were significantly associated with outcome. As regards ambulatory BP phenotypes, compared to patients with CH, the risk of HF hospitalization was not significantly higher in those with WUCH (HR 1.31, 95% CI 0.53–3.23), but it was significantly higher in those with ANRH (HR 2.29, 95% CI 1.14–4.59) and even higher in those with ARH (HR 4.02, 95% CI 1.92–8.43). The other variables reported in Table 1 did not attain statistical significance. Clinic and ambulatory BP were not evaluated because they are included in the definition of ambulatory BP groups. Pre-awakening morning systolic BP surge (high morning surge, HR 1.63, 95% CI 0.81–3.31) and nondipping for systolic BP (HR 1.60, 0.91–2.80) tended to be associated with outcome but did not attain statistical significance in this population in which HF subtypes were analyzed together. Antihypertensive drug classes were not associated with the outcome.

**Figure 3:**
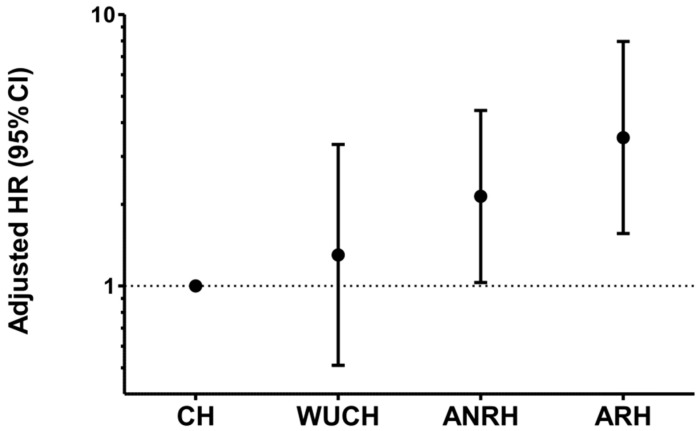
Adjusted hazard ratio (HR) and 95% confidence interval (CI) for heart failure hospitalization in patients with white coat uncontrolled hypertension (WUCH), ambulatory non-resistant hypertension (ANRH), and ambulatory resistant hypertension (ARH), when compared to those with controlled hypertension (CH). Adjustments included age, diabetes, left ventricular (LV) hypertrophy, asymptomatic LV systolic dysfunction, previous events, estimated glomerular filtration rate, left atrial enlargement, pre-awakening systolic BP surge and nondipping for systolic BP. Adjusted HR (95% CI) is 1.30 (0.51–3.32), 2.14 (1.03–4.43), and 3.52 (1.56–7.96) for WUCH, ANRH and ARH, respectively.

The results of multivariate Cox regression analysis are reported in Figure 3. We built a model including age, diabetes, LV hypertrophy, and asymptomatic LV systolic dysfunction that were significantly associated with outcome in univariate analysis, and forced into the model previous events, estimated glomerular filtration rate, LA enlargement, pre-awakening morning systolic BP surge and nondipping for systolic BP because of their potential and/or previously reported influence on the occurrence of HF hospitalization. After adjustment for the abovementioned covariates, compared to CH, the adjusted HR (95% CI) for HF was 1.30 (0.51–3.32), 2.14 (1.03–4.43) and 3.52 (1.56–7.96) in WUCH, ANRH, and ARH, respectively. If pre-awakening morning systolic BP surge, as a continuous variable, and nondipping for systolic BP, as a categorical variable, were replaced in the model by a categorical variable including three groups, that is, dippers with normal morning systolic BP surge, dippers with high morning systolic BP surge, and nondippers, the HR (95% CI) for HF in patients with WUCH, ANRH, and ARH remained substantially the same, that is, 1.25 (0.49–3.33), 2.09 (1.00–4.39), 3.48 (1.54–7.90), respectively.

## 4. Discussion

This study shows that, in the specific setting of elderly treated hypertensive patients, the risk of HF hospitalization was 2-fold and 3.5-fold higher in ANRH and ARH, respectively, when compared to CH.

Few studies have reported that ambulatory BP parameters are superior to clinic BP in predicting HF [25,26]. A single study evaluated the influence of ARH on the occurrence of HF [32] in hypertensive patients. In their remarkable investigation in the Japan Ambulatory Blood Pressure Monitoring Prospective study, Kario et al. [32] assessed 5839 hypertensive individuals aged 69 ± 12 years who were followed up for 4.5 ± 2.4 years. Among the studied subjects, 421 had ARH. During the follow-up, 67 HF hospitalizations occurred in the global population. After adjustment for covariates, compared to patients with CH, the HR (95% CI) for HF events was 2.24 (1.17–4.30) in individuals with ARH by using a 24-h BP threshold (130/80 mmHg) to define ambulatory BP phenotypes.

Despite some differences between the study by Kario et al. [32] and ours, such as population type and ethnicity, both point in the same direction, that is that patients with ARH have a substantially high risk to develop HF. At variance with the previous study [32], our results are specifically related to a hypertensive population older than 65 years.

In our study, patients with ANRH also had a higher risk of HF than patients with CH. However, in this group of patients, there is a margin for increasing antihypertensive therapy and reducing the risk of HF.

From a therapeutic point of view, the most difficult challenge is present in patients with ARH who are already taking three or more drugs. These patients require additional therapies to reduce BP values and the risk of HF and other cardiovascular outcomes. It has recently been reported that a reduction of 5 mmHg of systolic BP is associated with a 13% reduction in HF risk [39]. There is evidence that the addition of Spironolactone to patients with apparent resistant hypertension reduces BP to a greater extent than other antihypertensive drugs [40] and that it reduces both clinic and ambulatory BP [41]. Indeed, the addition of Spironolactone in patients with apparent resistant hypertension is now indicated in all recent guidelines [1,2,42,43]. Of our patients with ARH, recruited from 1992 to 2012, only 14% of them received Spironolactone.

Another pharmacological class with high potential in the treatment of resistant hypertension to reduce BP values and outcomes, particularly HF, is represented by glyphozines (or Sodium-Glucose Cotransporter 2 Inhibitors). In randomized trials, empaglifozin, canaglifozin, dapaglifozin, and ertuglifozin have reduced HF hospitalization by about 30% in diabetic subjects, the vast majority (>90%) of whom had also hypertension and were treated with antihypertensive drugs [44,45,46,47]. It has also been reported that glyfozines are associated with a reduction of systolic BP (about 4 mmHg) and diastolic BP (about 2 mmHg) when compared to placebo or active comparators [48]. Moreover, in the EMPA-REG BP trial [49], it has been shown that the addition of empaglifozin further reduces 24-h systolic/diastolic BP (about 4 mmHg/2 mmHg) irrespective of the number and type of antihypertensive drug used. A further reduction of daytime, nighttime, and 24-h BP by adding empaglifozin, versus placebo, in treated diabetic and hypertensive patients was also reported in the SACRA study [50]. Thus, considering the ability of glyphozines to further reduce BP in treated hypertensive patients and to reduce HF hospitalization in primary prevention in patients with diabetes and hypertension, they appear as valuable drugs for patients with uncontrolled BP and high risk of HF, such as those with ARH. Patients evaluated in the present study did not receive Sodium-Glucose Cotransporter 2 Inhibitors.

An additional drug class that could be helpful in the treatment of ARH is the angiotensin receptor neprilysin inhibitor. Indeed, it has been reported in some studies that angiotensin receptor neprilysin inhibitor significantly reduces both clinical and ambulatory BP when compared to placebo or active treatment [51,52,53,54]. If an angiotensin receptor neprilysin inhibitor should be used in ARH, therapy with angiotensin-converting enzyme inhibitor/angiotensin receptor blocker must be discontinued, and another antihypertensive drug should be added in addition to angiotensin receptor neprilysin inhibitor.

This study has some limitations. First, we evaluated only Italian patients and our results may not be applied to other ethnic groups. Second, we assessed only treated hypertensive patients older than 65 years and our data cannot be extrapolated to other types of hypertensive populations. Third, some of these patients were followed up in our outpatient clinic and part by their family doctors. Thus, BP control was not known in all the patients. Fourth, ambulatory BP data were obtained at baseline in all the patients, but only part of them underwent repeated ambulatory BP monitoring during time; thus, potential temporal changes and their relevance could not be evaluated. Fifth, in order to maintain adequate statistical power, we did not analyze HF subtypes separately. Sixth, we did not specifically design a study to estimate the risk associated with ARH, but this study is part of a prospective evaluation of the prognostic relevance of ambulatory BP parameters and other risk markers in our initially treated hypertensive patients.

Our study also has some strengths including (1) the use of ambulatory BP monitoring to define true resistant hypertension, (2) a fairly large sample size, (3) a large number of HF events, (4) adjustment of risk by means of a substantial number of covariates.

## 5. Conclusions

Our data show that, among elderly treated hypertensive patients, those with ARH are at a considerable higher risk of developing HF when compared to CH. New therapeutic strategies including drugs that have been shown to be effective in the primary prevention of HF and in reducing BP could lighten the burden of this outcome in arterial hypertension, and particularly in resistant hypertension, in the future.

## Figures and Tables

**Table 1:** Characteristics of study groups at baseline.

Parameter	CH	WUCH	ANRH	ARH
*n*	153	153	307	132
Age, years	71 (68–74)	72 (69–76)	71 (68–76)	72 (69–78)
Men, *n* (%)	62 (40)	55 (36)	134 (44)	53 (40)
Body mass index, kg/m^2^	27 ± 4	28 ± 4	27 ± 4 †	28 ± 3 ‡
Smokers, *n* (%)	21 (14)	10 (6)	29 (9)	13 (10)
Previous events, *n* (%)	11 (7)	22 (14)	28 (9)	14 (11)
Diabetes, *n* (%)	17 (11)	15 (10)	50 (16)	32 (24) *†
eGFR < 60 mL/min, *n* (%)	60 (39)	76 (50)	128 (42)	72 (54) *
LDL cholesterol, mg/dL	131 ± 28	123 ± 29	128 ± 29	122 ± 28
LV hypertrophy, *n* (%)	29 (19)	31 (20)	119 (39) *†	66 (50) *†
LA enlargement, *n* (%)	23 (15)	23 (15)	74 (24)	48 (36) *†
ALVSD, *n* (%)	6 (4)	8 (5)	13 (4)	9 (7)

ALVSD, asymptomatic left ventricular systolic dysfunction (ejection fraction < 50% at baseline); ANRH, ambulatory non-resistant hypertension; ARH, ambulatory resistant hypertension; CH, controlled hypertension; eGFR, estimated glomerular filtration rate; LDL, low-density lipoprotein; LA, left atrial; LV, left ventricular; WUCH, white coat uncontrolled hypertension. * *p* < 0.05 vs. CH; † *p* < 0.05 vs. WUCH; ‡ *p* < 0.05 vs. ANRH.

**Table 2:** Blood pressure values of study groups at baseline.

Parameter	CH	WUCH	ANRH	ARH
Clinic SBP, mmHg	130 ± 9	153 ± 10 *	159 ± 18 *†	160 ± 15 *†
Clinic DBP, mmHg	77 ± 8	87 ± 8 *	89 ± 10 *	88 ± 11 *
Daytime SBP, mmHg	121 ± 7	127 ± 7 *	144 ± 12 *†	146 ±13 *†
Daytime DBP, mmHg	72 ± 7	73 ± 6	80 ± 8 *†	79 ± 9 *†
Nighttime SBP, mmHg	111 ± 9	114 ± 9	132 ± 13 *†	137 ± 15 *†‡
Nighttime DBP, mmHg	63 ± 7	62 ± 7	69 ± 9 *†	70 ± 9 *†
24-h SBP, mmHg	118 ± 7	122 ± 6 *	140 ± 11 *†	143 ± 13 *†‡
24-h DBP, mmHg	69 ± 6	70 ± 6	77 ± 8 *†	76 ± 9 *†
MSBP surge, mmHg	11 ± 10	14 ± 10	14 ± 13	10 ± 13 †‡
DNMSBP surge, *n* (%)	52 (34)	52 (34)	72 (23)	26 (20) *†
DHMSBP surge, *n* (%)	13 (8)	26 (17)	68 (22) *	15 (11) ‡
NDSBP, *n* (%)	88 (57)	75 (49)	167 (54)	91 (69) †‡

ANRH, ambulatory non-resistant hypertension; ARH, ambulatory resistant hypertension; CH, controlled hypertension; DBP, diastolic blood pressure; DHMSBP, dippers with high morning systolic blood pressure (surge), that is, >23 mmHg as previously reported [26]; DNMSBP, dippers with normal morning systolic blood pressure (surge), that is, <23 mmHg as previously reported [26]; MSBP, morning systolic blood pressure (surge); NDSBP, nondippers for systolic blood pressure; SBP, systolic blood pressure; WUCH, white coat uncontrolled hypertension. * *p* < 0.05 vs. CH; † *p* < 0.05 vs. WUCH; ‡ *p* < 0.05 vs. ANRH.

**Table 3:** Therapy of study groups at baseline.

Parameter	CH	WUCH	ANRH	ARH
Diuretic, *n* (%)	74 (48)	100 (65) *	140 (46) †	117 (89) *†‡
Beta-blocker, *n* (%)	43 (28)	49 (32)	60 (19) †	58 (44) *‡
CCB, *n* (%)	47 (31)	48 (31)	85 (28)	80 (61) *†‡
ACE-I, *n* (%)	70 (46)	93 (61) *	143 (47) †	90 (68) *‡
ARB, *n* (%)	36 (23)	40 (26)	75 (24)	37 (28)
Alpha-blocker, *n* (%)	15 (10)	24 (16)	25 (8)	40 (30) *†‡
1 drug, *n* (%)	65 (43)	25 (16) *	90 (29) *†	0 (0) *†‡
2 drugs, *n* (%)	57 (37)	70 (46)	217 (71) *†	0 (0) *†‡
3–5 drugs, *n* (%)	31 (20)	58 (38) *	0 (0) *†	132 (100) *†‡
Aspirin, *n* (%)	39 (25)	39 (25)	79 (26)	46 (35)
Statin, *n* (%)	18 (12)	22 (14)	28 (9)	19 (14)

ACE-I, angiotensin-converting enzyme inhibitor; ANRH, ambulatory non-resistant hypertension; ARB, angiotensin receptor blocker; ARH, ambulatory resistant hypertension; CCB, calcium channel blocker; CH, controlled hypertension; WUCH, white coat uncontrolled hypertension. * *p* < 0.05 vs. CH; † *p* < 0.05 vs. WUCH; ‡ *p* < 0.05 vs. ANRH.

## Data Availability

The data underlying this article are available on reasonable request from the corresponding author.
